# Prevalence and Cognitive Bases of Subjective Memory Complaints in Older Adults: Evidence from a Community Sample

**DOI:** 10.1155/2014/176843

**Published:** 2014-04-27

**Authors:** Thomas Fritsch, McKee J. McClendon, Maggie S. Wallendal, Trevor F. Hyde, Janet D. Larsen

**Affiliations:** ^1^Parkinson Research Institute of the Wisconsin Parkinson Association, 945 North 12th Street, Suite 4602, Milwaukee, WI 53233, USA; ^2^Neurological Outcomes Center, Neurological Institute, University Hospitals Case Medical Center, 11100 Euclid Avenue, Cleveland, OH 44106, USA; ^3^Department of Psychology, Cardinal Stritch University, 6801 North Yates Road, Milwaukee, WI 53217, USA; ^4^Department of Psychology, John Carroll University, John Carroll Boulevard, University Heights, OH 44118, USA

## Abstract

*Objectives*. To estimate the prevalence of subjective memory complaints (SMCs) in a sample of community-dwelling, older adults and to examine cognitive bases of these complaints. * Participants*. 499 community-dwelling adults, 65 and older. *Measurements*. A telephone survey consisting of cognitive tests and clinical and sociodemographic variables. SMCs were based on subjects' evaluations and subjects' perceptions of others' evaluations. * Analysis*. Logistic regression was used to model the risk for SMCs as a function of the cognitive, clinical, and sociodemographic variables. We tested for interactions of the cognitive variables with age, education, and gender. *Results*. 27.1% reported memory complaints. Among the younger age, better objective memory performance predicted lower risk for SMCs, while among the older age, better memory had no effect on risk. Among the better-educated people, better global cognitive functioning predicted lower risk for SMCs, while among the less-educated people, better global cognitive functioning had no effect on SMC risk. When predicting others' perceptions, better objective memory was associated with lower risk for SMCs. *Conclusion*. Objective memory performance and global cognitive functioning are associated with lower risk for SMCs, but these relationships are the strongest for the younger age and those with more education, respectively. Age and education may affect the ability to accurately appraise cognitive functioning.

## 1. Introduction

Older adults often report that they are concerned about their memory and some bring these complaints to their primary care providers (PCPs). Estimates of the prevalence of subjective memory complaints (SMCs) vary but are generally high, ranging from approximately 25% to 50% [1]. Since a feeling of failing memory can be accompanied by psychological distress and worry, PCPs must be equipped to respond to questions about the meaning and relative importance of these complaints. For example, SMCs could have prognostic value in predicting objective memory decline and dementia [2, 3].

There is evidence that measures of subjective and objective memory are correlated, but the magnitude of the association is usually small [1]. Sociodemographic variables, such as age, education, and gender, also have been reported as predictors of SMCs. Older adults, women, and those with less education more often report SMCs than younger adults, men, and those with more education [1]. This may be because advancing age is associated with cognitive impairment; women, more often than men, report their health concerns; and those with more education may be better able to accurately appraise their cognitive functioning than those with less education [1, 4]. Another commonly reported finding is that depression or depressive symptoms are associated with SMCs [5]. This may be due to the tendency of persons with depression or depressive symptoms to exaggerate their deficiencies [6].

Some authors have pointed to the possible* moderating effects* of sociodemographic variables on the association between objective memory problems and SMCs [1, 4]. For example, older adults' assessments of their memory functioning may be influenced by a belief that memory declines with age, leading them to magnify any problems they do have. This effect may be independent of their objective memory functioning. On the other hand, younger adults may be less influenced by such an expectancy, resulting in more accurate appraisals of their own objective memory functioning. Similarly, adults with less education may be affected more by this kind of expectancy than adults with more education, regardless of their age. Thus adults with more education may be better able to appraise their own objective memory functioning. Empirically, these relationships would be shown through statistical interactions between variables assessing objective memory and sociodemographic variables. However, surprisingly few studies have directly tested for interaction effects between objective memory and sociodemographic variables to examine their impact on risk for SMCs.

Further, previous studies are limited by the sampling strategy. Studies with subjects drawn from memory clinics are biased in favor of finding a* higher* prevalence of SMCs because the respondents are more likely to have objective memory deficits. On the other hand, population-based studies may be biased in favor of finding a* lower* prevalence of SMCs because respondents are generally healthier and more willing to participate in research. However, in studies using telephone screening, persons with memory deficits might be less likely to refuse participation, thereby including a more representative sample of individuals having a range of memory abilities who are willing to participate in research. Thus, estimates of prevalence in a telephone study might be more accurate.

Importantly, relatively little is known about the* cognitive bases* of SMCs. While deficits in memory may well be the major source of SMCs, deficits in other cognitive domains may contribute to a feeling a failing memory. Attention, efficiency of information processing, and verbal skill each plays a unique role in the formation of memories. Thus, specific deficits in verbal functioning, attention, and processing speed could each independently increase the risk of SMCs. Declining global cognitive functioning could also contribute to the risk for SMCs. However, little is known about the relative and independent contributions of deficits in specific domains of cognitive functioning, as well as global cognitive functioning, to risk for SMCs.

Memory complaints have been assessed in a variety of ways. Some authors have used psychometrically validated scales (e.g., the Memory Functioning Questionnaire [7]) to assess the extent and frequency of memory complaints, but others have used single-item rating scales. We wondered whether there would be agreement between participants' beliefs about their own memory function and their perceptions of what others—such as family and friends—think about their memory function. Anecdotally, we know that persons with hearing loss often claim that the degree of loss is less than what family members perceive and report [8]. A similar phenomenon may occur when assessing SMCs from the vantage point of the subject and also from the vantage point of the subject's perceptions of what family members think. These two assessments of SMCs may agree or disagree, but little is known about this.

In the present research, we used telephone interviews to screen for SMCs in a sample of persons in the age of 65 and older living in Cuyahoga County, Ohio. We used two measures of SMCs. One focused on subjects' perceptions of their memory, and another focused on subjects' perceptions of evaluations by family members. We also assessed objective cognitive functioning with tests of episodic memory, verbal fluency, processing speed, and global cognitive functioning. Because we expected that most of our research volunteers would have self-awareness allowing them to encode instances of memory problems and have introspective access to that information, we predicted that deficits in objective memory would be associated with increased risk for SMCs. However, we predicted that there would not be an effect on risk for SMCs as a function of deficits in verbal fluency, processing speed, and global cognitive functioning, since we did not expect that subjects would attribute deficits in these domains to memory functioning.

We further predicted that there would be significant* interactive effects* between objective memory and the sociodemographic variables. Based on logic outlined earlier, we hypothesized that there would be statistically significant interactions between (1) age and memory, such that better objective memory would be associated with lower risk for SMCs in younger adults, but not in older adults; (2) education and memory, such that better objective memory would be associated with lower risk for SMCs in better-educated adults, but not in less-educated adults; and (3) gender and memory, such that objective memory would be associated with greater risk for SMCs in women than men. We did not predict that there would be significant interactions between the sociodemographic variables and other domains of cognitive functioning.

## 2. Methods

This study is based on data from the Cleveland Area Telephone Survey (CATS), a population-based study of cognitive aging and dementia initiated in 2002. In the CATS, telephone interviews were used to gather cognitive data on a sample of approximately 500 community-dwelling adults, age 65 and older, living in Cuyahoga County in Northeastern Ohio. The study was mainly designed to examine the feasibility of generating a representative sample for future epidemiologic studies involving assessment of memory and cognition in older adults.

### 2.1. Sampling

Study procedures associated with this project were reviewed and approved by the Institutional Review Board (IRB) for Human Investigation of University Hospitals of Cleveland (IRB 07-02-02). The IRB approved the study with a waiver of documented consent according to 45 CFR 46.117(c)(2).

Each potential subject first received a letter describing the project and soliciting participation. Potential subjects were told that they would soon receive a telephone call from a research assistant (RA) working at the University Memory and Aging Center, affiliated with University Hospitals of Cleveland and Case Western Reserve University. The letter outlined, in general, the following: (1) the purposes of the project; (2) its risks and benefits; (3) that study results would be reported in an aggregated form so that no person or persons could ever be identified in publications or reports; and (4) that the participants could withdraw from the study at any time. When the RA telephoned the potential subject and if the person agreed to participate, the study procedures, risks and benefits, and voluntary and confidential nature of the study were all described again. An RA used a script. If verbal consent was given, the subjects' willingness to participate in the research was recorded in a data manual by ID number and stored in a locked file cabinet. By contrast, if a subject indicated that he or she did not wish to participate, their “refusal” was also recorded. The person was thanked for their time, and the RA removed their name and any other identifying information from our list of potential participants.

The target population consisted of residents of households in Cuyahoga County with listed telephone numbers who were of age 65 and older in 2002. Cleveland is the largest city in Cuyahoga County. The population was limited to households with listed numbers for two reasons: (1) the households' addresses could be obtained so that we could send them a letter describing the study before calling them to conduct the telephone interview; (2) knowledge of the telephone number and the name of the householder listed in the telephone directory made it possible to select a sample of households that were known or expected to have at least one resident who was 65 or older. Households expected to have at least one person of age 65 or older were used to reduce the need for extensive screening for older persons that would be required if a random-digit-dialed survey of all households with telephones had been conducted. (Note that data for this study were collected in 2002-2003, when mobile phones were less prevalent. At this time, “land lines” were still in use to a great degree; thus our telephonic approach for sampling was reasonable at that time.)

A randomly selected list of households that were likely to meet our inclusion criteria was provided by* Survey Sampling, Inc.*—a leader in its field with over 25 years of experience.* Survey Sampling, Inc.* gathers data on age, education, race, gender, and income through contests, warranty cards, phone solicitation, and other means. For households with missing data, regression models are used to estimate whether or not there was someone of age 65 or older in the household.

Some time later, RAs, trained in basic interviewing techniques, neuropsychological test administration, data management, and subject confidentiality, telephoned the households. After a brief introduction, the RA asked if there was anyone of age 65 or older living in the household. If there was more than one person of age 65 or older in the household, the RA asked for the householder who was oldest, next oldest, next youngest, or youngest, depending on the number of persons of age 65 or older and a prespecified randomization scheme. After the selection of the subject, verbal consent was obtained and the RA proceeded with the survey.

Of the 1,897 households we attempted to contact, 350 were ineligible (e.g., no one of age 65 or older, undeliverable letter, or potential subject deceased or impaired too much). Of the remaining 1,547 households, interviews were conducted in 499 of them, yielding a response rate of 32.3%. This was a conservative estimate because we assumed that the 249 households where there was no answer after three calls may have contained eligible subjects. Our completion rate, indicating how successful we were in completing an interview when an eligible household was contacted, was 38.7%.

Before responding to the questions, participants were asked whether they had hearing impairments (*yes* was coded as 1, and* no* was coded as 0). Subjects who reported hearing impairments and who had hearing devices were encouraged to use them. The interviewer asked if there were others in the room or if a television or radio were on. If so, the subject was asked to go to a quiet room with a telephone to be interviewed.

Among subjects who participated in the study, the mean age was 75.7 years (SD = 6.4); 62.2% were women; and 83.0% were Caucasian (most of the minority participants were African Americans). The mean years of education attained by subjects in the study sample were 13.2 (SD = 3.0). Approximately 18% had less than 12 years of education, 36% had a high school diploma, and 45% had more than 12 years of education (values do not sum to 100% due to rounding).

According to data gathered for the 2000 US Census [9], 15% of persons living in Cuyahoga County were 65 years and older (*n* = 217,177). Among these, 60.8% were women and 48.0% were Caucasian. In terms of education, approximately 35% of residents of Cuyahoga County, of age 65 and older, had less than 12 years of education, 34% had a high school diploma, and 31% had more than 12 years of education. Thus, when comparing the older population in Cuyahoga County to our study sample, we see that our study sample overrepresented Caucasians and persons with higher education.

### 2.2. Measures

#### 2.2.1. Subjective Memory Complaints

We assessed subjective memory complaints with two questions. In the first, subjects were asked about their* own perceptions* of their memory: “would you say that you have a* serious problem* with your memory (coded as 2), that your memory is* somewhat of a problem* (coded as 1), or that it is* not much of a problem* (coded as 0)?” Subjects were then asked what they thought* others* think about their memory: “do other people find that you are* often forgetful* (coded as 2),* sometimes forgetful* (coded as 1), or* seldom if ever forgetful* (coded as 0)?”

#### 2.2.2. Objective Cognitive Performance

Global cognitive functioning was evaluated with a modified version of the Telephone Interview for Cognitive Status (TICS-m) [10]. This instrument was developed as a telephone version of the Mini-Mental State Examination (MMSE) [11]. The TICS-m tests function in several domains: concentration, orientation, memory, naming, comprehension, and abstraction. The TICS-m is reliable, with a high test-retest correlation over 18 months (*r* = .83) [12]. The TICS-m correlates highly with the MMSE (*r* = .80), indicating its validity as a measure of global cognitive functioning [12]. Because the TICS-m is heavily weighted towards assessing memory (20 of the 50 items are memory items), we modified the instrument to exclude the memory items. With the exclusion of the memory items, scores on the test could range from 0 to 30, with higher scores indicating better performance.

The Hopkins Verbal Learning Test (HVLT) is a list-learning and memory test [13]. The test consists of three free-recall trials of a 12-item list. A word-list is made up of 3 groups of 4 words belonging to different conceptual categories. A yes/no recognition task follows the free-recall tests. The HVLT has 6 alternate forms. In the present study, we used Form 1. The HVLT is easy to administer and can be used in normal and clinically impaired populations, such as Alzheimer's disease (AD). For total recall, test-retest reliability, measured over a 9-month interval, has been reported as good (*r* = .50) [14]. Validity has been shown through correlations with the* Logical Memory* subtests (immediate and delayed recall) of the Wechsler Memory Battery (*r* = .75 and *r* = .77, respectively) [15]. Scores on the test range from 0 to 36, with higher scores indicating better performance.

Verbal fluency was assessed through “animal naming,” in which subjects are asked to name as many animals as possible in one minute [16]. The test assesses verbal production and language and is sensitive to semantic retrieval deficits. Verbal fluency also assesses attention and executive functioning. Verbal fluency scores reflect the total number of animals named in one minute, with high scores indicating better performance.

In the “Timed Months of the Year Backwards” (TMYB) test [17], subjects are asked to say the months of the year backwards, starting with December. Response latencies are measured with a stopwatch. The test is a measure of central processing speed. It taps constructs such as attention, executive functioning, and working memory. According to research by the authors of the test, reliability, assessed with a test-retest correlation obtained one week to ten days later, is high, with *r* = .90 [17]. Construct validity is also high, when assessed with correlations between TMYB and measures of simple reaction time (*r* = .52) and choice reaction time (*r* = .51) and the Trails B test (*r* = −.45) [17]. The maximum time allowed to complete the task is 75 seconds. Lower scores reflect better performance.

#### 2.2.3. Self-Reported Clinical History

Subjects were asked to report whether a doctor had ever diagnosed them with a memory problem, and they were also asked whether parents, brother(s), sisters(s), or children had ever been diagnosed with a condition causing a memory problem. Responses were coded as 1 for* yes* and 0 for* no* for both questions.

### 2.3. Statistical Analysis

Because few people reported that they had* a serious memory problem*, while relatively more reported that they had* somewhat of a problem*, these two categories were combined, resulting in a dichotomous outcome variable (1 = SMC present, 0 = SMC absent). The same recoding was done for the variable reflecting how subjects felt that* others* would evaluate their memory. Binary logistic regression analysis was used to model the presence of SMCs as a function of several predictor variables: cognitive variables (episodic memory function [HVLT]; verbal fluency [animal naming]; processing speed [TMYB]); self-reported clinical history of memory problems (physician's diagnosis; family history); and sociodemographic variables (age, education, and gender). Interactions between the cognitive variables with age, education, and gender were also examined. Significant predictors were centered about their respective means to make the regression coefficients for the cognitive variables, age, and education more meaningful.

## 3. Results

The prevalence of memory complaints, stratified by age and education level, is shown in Table 1. Overall, 26.5% of subjects reported that their memory is* somewhat of a problem*, while 0.6% reported that they have* a serious problem* with their memory. Persons 75 years and older with 12 years of education had the highest reported prevalence of SMCs (35.5%), while persons of age 65 to 74 with 13 or more years of education had the lowest reported prevalence of SMCs (20.2%).

In terms of others' perceptions, 36.0% of subjects indicated that others thought the subjects were* sometimes forgetful*, while 2.3% reported that others thought they were* often forgetful*. Persons of age 65 to 74 with less than 12 years of education most often reported that others thought the subject had a memory problem (48.6%), while persons of age 75 and older with 13 or more years of education least often reported that others thought the subject had a memory problem (31.3%).

Interestingly, Table 1 also shows that those who were older less often reported a family history of memory loss, while increasing education was associated with reports of decreasing memory impairment diagnoses.

In Table 2, we present results of a logistic regression analysis, with subjects' subjective perceptions of their memory as the dependent measure. Predictor variables in the model were sociodemographic and clinical variables, cognitive measures, and terms for the interaction of cognitive variables with age, education, and gender. Nonsignificant interaction terms were removed from the model.

Table 2 indicates significant main effects of education, hearing ability, a diagnosed memory problem, global cognitive function (TICS-m), and memory (HVLT) on SMCs. However, these main effects were qualified by significant interactions. The memory × age interaction was significant (OR = 1.007, 95% CI = 1.001–1.012). Among the younger-old, better objective memory performance was associated with lower risk for SMCs, while among the older-old better memory had no effect on risk. The TICS-m × education interaction was also significant (OR = .976, 95% CI = .940–.995). Among those with more education, better global cognitive functioning was associated with lower risk of a SMC, while among those with less education, better global cognitive functioning had no effect on SMC risk. Other interactions were not significant.

**Figure 1:**
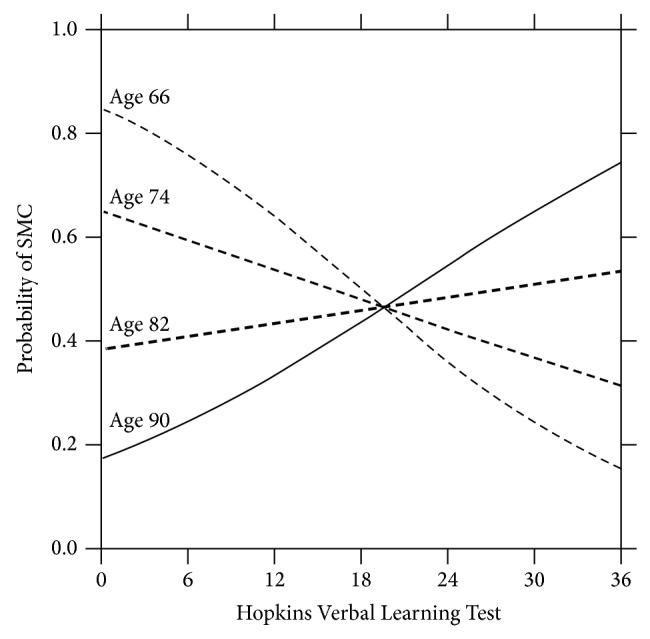
Probability of subjective memory complaints as a function of Hopkins Verbal Learning Test (HVLT) by selected years of age. (HVLT range of scores = 0 to 36; higher scores indicate better performance.) The Figure shows that objective memory performance was related to lower risk for SMCs, but these relationships were strongest for the younger-old.

**Figure 2:**
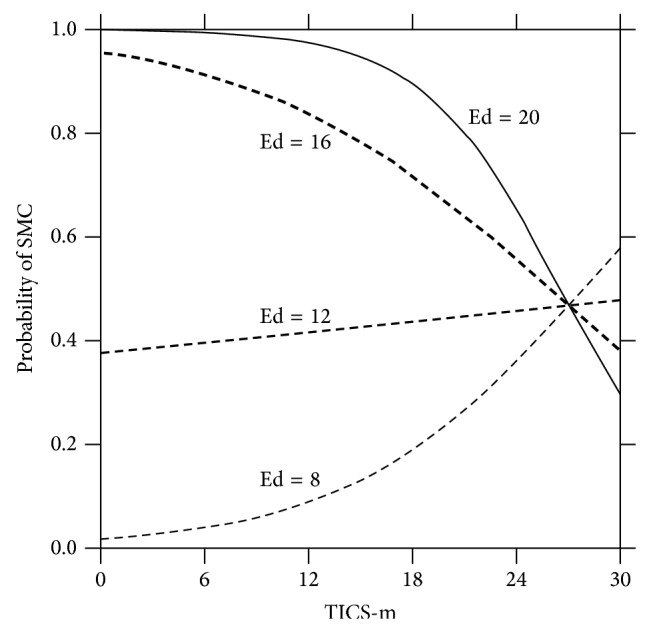
Probability of subjective memory complaints as a function of TICS-m by selected years of education. (TICS-m range of possible scores = 0 to 30; higher scores indicate better performance.) The figure shows that global cognitive functioning was associated with a lower risk for SMCs for those with higher levels of education.

Figures 1 and 2 show these complex relationships in graphical form. In Figure 1, we depict the chances of reporting a SMC as a function of the interactions between age and performance on our memory test, the HVLT. The specific ages shown in Figure 1 represent the range of ages in our study (66 through 90) and two frequently occurring ages in our data set (74 and 82).

The X-axis represents memory performance, with higher scores indicating better memory performance and lower scores indicating relatively poorer performance. Figure 1 shows that, depending on one's age, objective memory performance on our memory test predicted the chances of participants reporting a subjective memory complaint. For those with good memory (e.g., a score of 30 on the HVLT), the chances of reporting a subjective memory complaint were lower for those who were younger (e.g., 66 or 74) than for those who were older (e.g., 82 or 90). However, the pattern reverses quite dramatically for people who, when tested, had poor objective memory performance. For those with a very low memory score (e.g., 6 on the HVLT), the probability of reporting a memory complaint was greater for those who were younger (66 or 74) than for those who were older.

In Figure 2, the X-axis depicts performance on the TICS-m, our measure of overall cognitive ability. Figure 2 shows in graphical form that poor overall cognitive functioning—for example, a score of 6 on the TICS-m—was associated with vast differences by educational level in the probability that a subject would report a subjective memory complaint.

For example, for a person who completed 8 years of education and had a score of 6 on the TICS-m (meaning their cognitive functioning was very poor), the probability that they would report having a memory complaint was very low. By contrast, for those who completed relatively higher levels of educational attainment (e.g., 16 years, i.e., college graduates) and also had poor overall cognitive functioning (e.g., 6 on the Tics-m), the chances that they would report having a memory problem were far higher.

For persons whose cognitive performance was relatively better (e.g., 24 on the TICS-m), educational attainment level mattered less in determining whether a person would report a subjective memory complaint. Thus, for those who were cognitively intact, their educational attainment level was far less important in determining differences in the chances of reporting a memory complaint. To summarize, for those with poor overall cognitive functioning, educational attainment level greatly influenced the chances of reporting a memory complaint but for those with good cognitive functioning educational level had little or no influence on memory complaints.

In Table 3, we present results of a second logistic regression analysis,where participants' perception of others' beliefs about the participants' memory functioning was the dependent measure. None of the interactions of the cognitive and sociodemographic variables were significant in this model, so they were removed.

Table 3 indicates that better performance on the HVLT was associated with decreased odds of expectations that others would report memory impairments in our research volunteers. All other terms in the model were not significant.

## 4. Discussion 

There are three major findings emerging from the present research. First, in a sample of community-dwelling, older adults, the prevalence of SMCs was high, greater than 25%. This value is generally consistent with those reported in population-based studies [1] but lower than those reported in many clinic-based studies [18].

Second, we documented important statistical interactions between some of the cognitive and sociodemographic variables. Specifically, we found that objective memory performance was related to lower risk for SMCs, but these relationships were strongest for the younger-old. Global cognitive functioning was associated with a lower risk for SMCs for those with higher levels of education. This may be because, in assessing their own memory functioning, the older-old and those with less education tend to be influenced by a belief that memory declines with age, leading these subject groups to magnify any problems they may have had. This effect was independent of their objective memory functioning. On the other hand, the younger-old and the adults with more education may have been less influenced by such an expectancy, and therefore they were better able to* appraise* their own objective memory functioning [1, 4]. Importantly, performance on measures of verbal fluency and processing speed did not affect the odds of SMCs. While most of our research volunteers may have had sufficient self-awareness to encode instances of deficits in these cognitive domains, and while they may have had introspective access to that information, they may not have linked these deficits to problems with memory, specifically. Also, verbal fluency and processing speed did not interact in any way with age, education, or gender in each of the models we studied.

Third, the effect of objective memory ability on memory complaints seems to vary depending on whether subjects report on their own subjective experience when considering their memory functioning, versus when they consider how others evaluate their memory. Specifically, we only observed significant interactions between sociodemographic and cognitive variables when subjects reported on their own perceptions but not when they reported on perceptions of others' evaluations.

Our study has certain limitations. We used a novel technique for identifying and contacting a sample of elders in our catchment area, designed to reduce biases commonly found in clinic- and population-based studies. However, while comparisons between our study sample and 2000 Census data from Cuyahoga County showed that the groups were similar in terms of age and gender distribution, our study sample was comprised of more self-identified Caucasians than African Americans or other minorities and more persons with higher educational attainment levels. Also, our response rate was relatively low (about 33%). This response rate is lower than that reported in many random-digit-dialed phone surveys, which typically obtain rates ranging from 60 to 64% [19]. Another problem related to sampling concerns those households with unlisted numbers. Those with unlisted numbers are known to be younger and of lower socioeconomic status (SES) than those with listed numbers. The SES bias presumably occurs because persons with low SES are more mobile and thus more likely to be unlisted. However, mobility would presumably be low among our sample members due to their age, and thus there might not be an SES bias. Each of these limitations, individually, or together, may have biased results towards an underestimate of the prevalence of SMCs.

Other studies have used psychometrically validated scales to measure SMCs [7], whereas we relied on simple Likert-type ratings. However, even with this rudimentary measure, we were able to show associations with objective memory abilities. The associations held even when controlling for important clinical variables such as family history and/or physician diagnosis of memory problems. The fact that results were dependent on whether subjects took the vantage point of self versus other is particularly interesting. Apparently, subjects are able to keep the sources of concerns about their memory seperate (their own versus others). This demonstrates flexibility in an important metacognitive ability.

Our study did not include a measure of depression. Our reasoning centered on the fact that interviews were relatively long, lasting from 30 to 40 minutes. We did not want to create too much subjective burden on subjects, risking our researcher/subject rapport, possibly resulting in a loss of the vital neuropsychological data.

Yet, from a clinical perspective, we have noted that PCPs have improved in their recognition of depressive symptoms in routine office visits but many are not aggressive in characterizing memory loss as a possible outcome of depression. We believe that when a person has an SMC that causes them distress, they should start to address the problem by visiting their PCP and describing their concerns. If the cause of the complaint (including depression) is not found or treated, then consulting a specialist (usually a general neurologist) is a reasonable next step. At the typical PCP visit, probing for depression (if it is not reported by the patient) would seem reasonable as depression is correlated with many health problems and assessing its presence does not consume much office time.

Further, while studies have indicated that depression or depressive symptoms are correlated with SMCs, with zero-order correlations ranging from .30 to .36 [20], the effect of adding depression to the equation depends not only on its correlation with SMCs but also very much on how strongly it is correlated with our measures of memory (HVLT) or global cognitive functioning (TICS-m). Since studies tend to support the idea that both memory and global cognitive functioning are not correlated very strongly with depression among the aged [21], including depression as a variable in our statistical models is not likely to alter the effect of memory or cognitive ability.

Subjective memory complaints occur with frequency in the population. Because they were a source of concern for participants and are likely to be of concern to many older healthy adults in the general population, it becomes vital to understand their origins and importance. Future studies should continue to examine the basis of these complaints as well as the modifying effects of contextual variables such as sociodemographic factors, in an effort to more easily determine which complaints reflect a real problem and which represent needless worry on the part of a patient.

## Figures and Tables

**Table 1:** Prevalence of subjective memory complaints, family history of memory problems, and diagnosis of memory disorder (*n*=499)^*^.

	Ages 65–74			Ages 75+			Total group
	0–11 yrs ed. % (*n*)	12 yrs. ed. % (*n*)	13+ yrs. ed. % (*n*)	0–11 yrs ed. % (*n*)	12 yrs. ed. % (*n*)	13+ yrs. ed. % (*n*)	% (*n*)
Subjective memory complaints							
Not much of a problem	68.6 (24)	70.6 (60)	79.8 (99)	76.4 (42)	64.5 (60)	74.5 (73)	72.9 (363)
Somewhat of a problem	28.6 (10)	29.4 (25)	20.2 (25)	23.6 (13)	34.4 (32)	25.5 (25)	26.5 (132)
A serious problem	2.9 (1)	0.0 (0)	0.0 (0)	0.0 (0)	1.1 (1)	0.0 (0)	0.6 (3)
Others' perceptions of your memory							
Seldom if ever forgetful	51.4 (18)	56.6 (47)	61.7 (74)	63.6 (35)	60.9 (53)	68.8 (66)	61.8 (299)
Sometimes forgetful	45.7 (16)	38.6 (32)	38.3 (46)	32.7 (18)	36.8 (32)	31.3 (30)	36.0 (174)
Often forgetful	2.9 (1)	4.8 (4)	0.0 (0)	3.6 (2)	2.3 (2)	0.0 (0)	2.3 (11)
Any family members with memory problems?							
No	77.1 (27)	76.5 (65)	76.9 (93)	90.6 (48)	87.9 (80)	83.0 (78)	81.9 (399)
Yes	22.9 (8)	23.5 (20)	23.1 (28)	9.4 (5)	12.1 (11)	17.0 (16)	18.1 (88)
Were you ever diagnosed with memory problem?							
No	94.3 (33)	96.5 (83)	99.2 (120)	96.3 (52)	97.8 (89)	100 (94)	98.0 (479)
Yes	5.7 (2)	3.5 (3)	0.8 (1)	3.7 (2)	2.2 (2)	0.0 (0)	2.0 (10)

^*^Totals for response to individual questions may not sum to 499 due to missing data.

**Table 2:** Logistic regression predicting memory complaint (coded as 1) versus no complaint (coded as 0) as a function of sociodemographic, clinical, and cognitive variables (*n* = 455). Also included in this model are significant interaction terms between age and memory as well as education and global cognitive functioning.

Variable	Odds ratio	95% CI	*P* value
Age	0.872	0.775–0.981	0.023
Education	2.442	1.119–5.328	0.025
Gender	1.089	0.665–1.782	0.735
Race	1.040	0.556–1.946	0.901
Hearing	2.209	1.401–3.482	0.001
Family history	1.560	0.902–2.698	0.111
Memory diagnosis	5.781	1.064–31.417	0.042
TICS-m	1.506	1.047–2.168	0.027
HVLT	0.573	0.374–0.877	0.010
Verbal fluency	0.992	0.939–1.047	0.771
Processing speed	1.003	0.978–1.028	0.829
Age × HVLT	1.007	1.001–1.012	0.018
Education × TICS-m	0.967	0.939–0.995	0.023

For gender, 0 = female, 1 = male. For race, 0 = minority, 1 = caucasian. For hearing, 0 = no impairment, 1 = impairment. For family history, 0 = no family history, 1 = family history.

TICS-m = Modified Telephone Interview for Cognitive Status (memory items were deleted from the scale). HVLT= Hopkins Verbal Learning Test.

**Table 3:** Logistic regression predicting other's perceptions that subjects have a memory complaint (coded as 1) versus no complaint (coded as 0) as a function of sociodemographic, clinical, and cognitive variables (*n* = 443).

Variable	Odds ratio	95% CI	*P* value
Age	0.987	0.953–1.021	0.435
Education	0.957	0.884–1.036	0.281
Gender	0.844	0.539–1.321	0.459
Race	1.265	0.714–2.242	0.420
Hearing	1.424	0.933–2.175	0.101
Family history	0.862	0.516–1.440	0.570
Memory diagnosis	3.505	0.667–18.419	0.138
TICS-m	1.048	0.952–1.153	0.338
HVLT	0.962	0.926–1.000	0.047
Verbal fluency	1.021	0.974–1.070	0.395
Processing speed	1.017	0.995–1.040	0.131

For gender, 0 = female, 1 = male. For race, 0 = minority, 1 = caucasian. For hearing, 0 = no impairment, 1 = impairment. For family history, 0 = no family history, 1 = family history.

TICS-m = Modified Telephone Interview for Cognitive Status (memory items were deleted from the scale). HVLT = Hopkins Verbal Learning Test.
